# Costs and Cost-Effectiveness of a Mobile Phone Text-Message Reminder Programmes to Improve Health Workers' Adherence to Malaria Guidelines in Kenya

**DOI:** 10.1371/journal.pone.0052045

**Published:** 2012-12-18

**Authors:** Dejan Zurovac, Bruce A. Larson, Raymond K. Sudoi, Robert W. Snow

**Affiliations:** 1 Malaria Public Health Cluster, KEMRI-Wellcome Trust–University of Oxford Collaborative Programme, Nairobi, Kenya; 2 Centre for Tropical Medicine, Nuffield Department of Clinical Medicine, University of Oxford, Oxford, United Kingdom; 3 Center for Global Health and Development, Department of International Health, Boston University, Boston, Massachusetts, United States of America; Swiss Tropical & Public Health Institute, Switzerland

## Abstract

**Background:**

Simple interventions for improving health workers' adherence to malaria case-management guidelines are urgently required across Africa. A recent trial in Kenya showed that text-message reminders sent to health workers' mobile phones improved management of pediatric outpatients by 25 percentage points. In this paper we examine costs and cost-effectiveness of this intervention.

**Methods/Findings:**

We evaluate costs and cost-effectiveness in 2010 USD under three implementation scenarios: (1) as implemented under study conditions in study areas; (2) if the intervention was routinely implemented by the Ministry of Health (MoH) in the same areas; and (3) if the intervention was scaled up nationally. Under study conditions, intervention costs were 19,342 USD, of which 45% were for developing and pretesting text-messages, 12% for developing text-message distribution system, 29% for collecting health workers' phone numbers, and 13% were costs of sending text-messages and monitoring of the system. If the intervention was implemented in the same areas by the MoH, the costs would be 28% lower (13,920 USD) due to lower costs of collecting health workers' numbers. The cost of national scale-up would be 97,350 USD, and the majority of these costs (66%) would be for sending text-messages. The cost per additional child correctly managed was 0.50 USD under study conditions, 0.36 USD if implemented by the MoH in the same area, and estimated at only 0.03 USD if implemented nationally. Even if the effect size was only 5% or the cost on the national scale was 400% higher than estimated, the cost per additional child correctly managed would be only 0.16 USD.

**Conclusions:**

A simple text-messaging intervention improving health worker adherence to malaria guidelines is effective and inexpensive. Further research is justified to optimize delivery of the intervention and expand targets beyond children and malaria disease.

## Introduction

The failure of health workers to manage patients according to national malaria guidelines remains a barrier to effective implementation of malaria case-management policies and provision of quality of care across Africa [1]–[4]. Several complex, labor intensive and expensive interventions, such as high-quality in-service training, supportive supervision of health workers, audit and feedback, and quality improvement schemes, have been suggested as interventions to improve drug use and adherence to guidelines in developing countries [5], [6]. More recently, the possibility of using mobile health technologies as simple, inexpensive but effective interventions for behavior change that could be easily replicated in weak health systems in Africa has been proposed [7], [8].

One such intervention tested in a randomized controlled trial in Kenya showed that simple text-message reminders sent regularly to health workers' personal mobile phones substantially improved management of pediatric outpatients in accordance with national malaria guidelines [9]. The quantitative effects of the intervention and qualitative investigations of health workers' perceptions have been presented elsewhere [9], [10]. Here we examine costs and cost-effectiveness of this intervention.

## Methods

We evaluate costs and cost-effectiveness under three different implementation scenarios: (1) as implemented in the trial under study conditions in the study districts; (2) if the basic intervention was implemented by the Ministry of Health in the same study locations under routine, non-study conditions where specific study related costs are removed; and (3) if the intervention was expanded to a national scale in Kenya and implemented entirely by the Ministry of Health.

All costs are financial costs reported in 2010 U.S. Dollar values (USD). Costs are evaluated from the perspective of the program implementer. For Scenario 1, costs are actual financial costs for implementing the study by the research programme. Based on the experience gained in implementing the study and improvements in access to low-cost bulk text-messaging systems that are now available in Kenya, costs for Scenarios 2 and 3 are based on estimates of resources needed and associated costs for the Ministry of Health. We evaluate the sensitivity of results for each scenario to increases in implementation costs or reductions in intervention effectiveness.

### Scenario 1 – The text-messaging intervention under the trial conditions in study areas

The cluster randomized controlled trial was undertaken between March 2009 and May 2010 at 107 rural government health facilities in two malaria endemic areas in Kenya (Greater Kisii/Gucha and Greater Kwale). The trial is registered with Current Controlled Trials, ISRCTN72328636 [9]. The detailed description of study areas and intervention characteristics was provided elsewhere [9], [10]. The intervention was a one-way communication of text-message reminders on paediatric malaria case-management accompanied by “motivating” quotes. The messages were sent to personal mobile phones of 119 health workers performing outpatient consultations at 54 intervention facilities in study areas.

The intervention development process and subsequent implementation included the following activities. First, the content, order, frequency and duration of the text-messages were developed over 5 days in partnership between researchers and policy makers of Kenyan Ministry of Health's Division of Malaria Control. The key messages addressed recommendations from the national malaria guidelines and training manuals valid at the time of the study, which recommended presumptive treatment of childhood fevers with artemether-lumefantrine (AL) and related AL dosing, dispensing and counseling tasks [11], [12]. During the same activity, understanding of text-messages was pretested during two rounds of individual interviews with 20 health workers from health facilities in neighboring study districts. In total, 10 different malaria text-messages were selected as part of this process. During the implementation of the study, messages were sent for 5 working days a week (two messages daily at 9am and 2pm). These messages were repeated every week during the 6-month intervention period.

Second, a computerized distribution system was developed on a desktop server interfaced with the network of a local mobile service provider through a global system for mobile communication modem. The system ensured automated delivery of text-messages according to a pre-determined list of phone numbers, timing of message transmission, and content of the text-message. The system was developed by an information technology specialist, who also provided maintenance support during the implementation period. The performance of the distribution system was tested in two rounds: first on 40 mobile phone numbers during 6 weeks of testing, and then on 120 recipients during one week. Third, the mobile phone numbers of all health workers at study facilities who were recipients of the intervention during the trial were collected by study teams during a health facility survey.

Finally, the intervention was implemented between May 4 and October 30, 2009, when 33,361 text-messages were sent to 119 health workers on 150 phone numbers (31 health workers had more than one number). During this period, the delivery of text messages was monitored by a research assistant using the computerized system developed by the information technology specialist.

### Scenario 2 - The text-messaging intervention if implemented under routine conditions in study areas

Under this scenario, all components of the intervention development and delivery would have remained the same as under the trial conditions with one exception, the collection of health workers' mobile phone numbers. As part of the trial, collecting these numbers was integrated into the health facility survey used to establish baseline case-management practices in study districts. If the Ministry of Health implemented this intervention, district supervisors would be required to update and verify already existing lists of health workers' phone numbers by district. The district supervisors would then provide the list to the intervention implementers at the national level. Considering this scenario is relevant because the relatively complex and expensive health facility surveys completed during the trial accounted for almost 30% of total costs of the Scenario 1. Such costs would fall substantially if the intervention was implemented by the Ministry of Health under routine, non-study conditions.

### Scenario 3 - Scaling up to the national level

If the Ministry of Health (MoH) integrated this intervention into national policy, and implemented the intervention at a national scale, two specific modifications to the basic intervention package would be required. First, Kenya has revised the national malaria outpatient treatment guidelines since 2010 and now recommends universal parasitological testing with microscopy or rapid diagnostic tests and adherence to diagnostic test results. Therefore, a process of revision of text-messages and its field pretesting would be required to incorporate changes in national guidelines. Second, scaling the intervention to 149 districts supporting approximately 20,000 health workers at 5,367 health facilities countrywide would require an automated distribution system able to support relatively high volume of messages sent within short time periods (twice daily, five times a week, for six months). We have considered here the standard way bulk SMS services are delivered in Kenya by network-authorized service providers already used to undertaking large-scale text-messaging campaigns, such as service advertisements or employee notifications. Finally, we have also incorporated costs for the development and maintenance of a web-based system for monitoring the delivery of the intervention for the MoH.

### Cost-effectiveness

For each of three scenarios we calculated costs of the intervention as the direct financial costs (USD 2010) for implementing the scenario. For Scenario 1, the full costs of implementing the study come directly from the financial records for the study. For Scenario 2, program implementation costs are adjusted to exclude costs from Scenario 1 that were for research purposes but would not be included if the intervention was implemented by the government as routine practice. Personnel costs are also based on standard government employee salary and benefits information for the staff implementing the intervention. For Scenario 3, we extrapolated resource needs from the study sites to the national level based on the number of districts in the country, estimates of the number of health workers receiving the messages, and commercial rates for bulk messaging.

For effectiveness information for Scenarios 1 and 2, we use the results of the cluster randomized controlled trial to estimate the additional number of febrile children correctly managed according to national guidelines due to the intervention. The intention-to-treat analysis showed that correct AL management, defined as a child treated according to national malaria treatment guidelines, improved immediately after the intervention and this improvement was maintained 6 months later with nearly equal effect sizes (23.7 and 24.5 percentage points respectively).

Thus, the additional number of children correctly managed by the intervention was estimated by multiplying annual number of sick children at the intervention facilities by the proportion of febrile children at the same facilities. The annual number of children was extracted from the outpatient registers at intervention facilities while the proportion of febrile children at the same facilities was imputed based on data collected during the health facility surveys used to evaluate the intervention. In total, 153,379 children in the study sites required correct management for malaria during one year. As a base case, we assume that 25% more children with fever were correctly managed according to treatment guidelines in the intervention sites as compared to the control sites based on the intention-to-treat analysis during the six-month trial and during the six-months following the end of the trial [9]. Based on these figures, 38,345 additional children were correctly managed in the study sites due to the intervention (38,345 = 0.25*153,379).

For Scenario 3, the annual number of febrile children requiring correct management for malaria was obtained from the latest national estimates of febrile children presenting to public health facilities in Kenya [13]. On the national scale, the annual number of febrile children presenting to public health facilities in 2007 was estimated to be about 11.8 million. Applying 25 percentage points effect size of the intervention at the national level, an annual number of febrile children correctly managed due to intervention would be about 3 million children.

Given the costs and effectiveness of the intervention estimated and modeled for each scenario, the average cost per additional child correctly managed is calculated as:
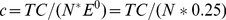
(1)where c is the cost per child correctly managed for each scenario, TC is the total costs of implementing the intervention for each scenario, N is the total number of children during a year needing correct management, and E^0^ is the effect size as a proportion (E^0^ = 0.25 is the base case assumption).

### Sensitivity analysis

Given the recent policy shift in Kenya from presumptive treatment for malaria to more complex management based on diagnostic tests and then treating according to test results, we consider the sensitivity of the results to higher program costs (a higher TC for each scenario) and a lower effect size. Conceptually, an increase in costs of the program is equivalent to a proportional reduction in the effect size. In the sensitivity analyses reported, we considered changes in effect size from 0.25 to 0.20, 0.15, 0.10, and 0.05, which are equivalent to increases in total costs of 25%, 67%, 150%, and 400% if the effect size remains 0.25.

### Ethical approval

Written informed consent was obtained from all health workers and caregivers of sick children during the trial and the study protocol was approved by the University of Oxford (OXTREC No 3808) and Kenya Medical Research Institute (SSC No 1329).

## Results

Table 1 shows the actual annual costs of developing and implementing the intervention in the study area under the trial conditions (Scenario 1) and estimated costs if the intervention was implemented under the routine conditions in the same area (Scenario 2). Table 2 estimates the annual cost if the intervention was scaled to a national level. Table 3 summarizes cost-effectiveness results. Appendix S1 provides further details of information and assumption included in the cost information for each Scenario.

**Table 1:** Costs for Scenarios 1 and 2.

	Scenario 1 (USD)	Scenario 2(USD)
**1. DEVELOPMENT OF INTERVENTION**		
A. Development and refinement of text-messages		
Senior researcher (5 days at full cost to employer)		
Research assistant (5 days at full cost to employer)		
Review of messages by 5 research officers (2 hours each, full cost)		
Review of messages and input from 2 DOMC officers (4 hours each)		
**Total labor**	3,217	3,217
B. Pretesting of messages with 20 HWs in non-study districts (2 rounds)		
Mileage cost transport	2,620	2,620
Salary (research assistant and driver, each for 20 working days)	1,569	1,569
Subsistence (research assistant and driver, each for 28 days)	1,391	1,391
**2. DEVELOPMENT OF DISTRIBUTION SYTEM**		
Consultation fee (for development and maintenance of the system)	1,005	1,005
Project computer (1 desktop)	938	938
Cost of 2 modems	177	177
Purchase of postpaid phone number for distribution	93	93
Airtime for testing distribution system	134	134
**3. COST OF COLLECTING HWs PHONE NUMBERS**		
Scenario 1. Study conditions		
Vehicle costs for traveling to facilities	2,682	
Research assistant and driver (full cost to employer, 20 working days each)	1,569	
Traveling subsistence (research assistant and driver, 28 days each)	1,391	
Scenario 2. Routine conditions – use of DHMTs to collect phone numbers		
District Public Health Nurses (7 days, full cost to employer)		193
Airtime for updating health workers' phone numbers		27
**4. IMPLEMENTATION COST**		
Total cost of sending text-messages	1,135	1,135
Research assistant (28 days, full employer costs)	1,423	1,423
**Total Cost**	19,342	13,920

DOMC = Division of Malaria Control; HW = health worker; DHMT = District Health Management Team.

**Table 2:** Costs for national scale-up (Scenario 3).

	Scenario 3 (USD)
**1. DEVELOPMENT OF INTERVENTION**	
A. Development and refinement of text-messages	
Senior consultant (5 days at full cost to employer)	
Division of Malaria Control Officers (5 officers, 5 days each, full cost to employer)	
Review of messages and input from 2 DOMC officers (4 hours each, full cost to employer)	
**Total labor**	5,463
B. Pretesting of messages with 20 health workers (2 rounds)	
Mileage cost transport	1,242
Salary (2 DOMC Officers and driver, each for 20 working days)	4,691
Subsistence (2 DOMC Officers and driver, each for 28 days, government per-diem rates)	7,823
**2. DEVELOPMENT OF DISTRIBUTION SYTEM**	
Development and maintenance of the system	7,177
Project computer (1 desktop)	938
**3. COST OF COLLECTING HWs PHONE NUMBERS**	
District Public Health Nurses (149 districts, 1 day/nurse/district, full cost to employer)	4,111
Airtime to update health workers' phone numbers	1,333
**4. IMPLEMENTATION COST**	
Total cost of sending text-messages (0.0124 USD per message full cost)	64,572
**Total Cost**	97,350

DOMC = Division of Malaria Control; HW = health worker.

**Table 3:** Cost-effectiveness and sensitivity analyses.

	Scenario 1	Scenario 2	Scenario 3
Costs (USD 2010)	19,342	13,920	97,350
Number of febrile children requiring correct management	153,739	153,739	11,821,000
Intervention effect (proportion of additional children correctly managed)	0.25	0.25	0.25
Additional number of febrile children correctly managed	38,435	38,435	2,955,250
Cost per additional febrile child correctly managed	0.50	0.36	0.03
Sensitivity Analysis (either/or)			
Effect = 0.20, 25% Cost increase	0.63	0.45	0.04
Effect = 0.15, 67% Cost increase	0.84	0.60	0.05
Effect = 0.10, 150% Cost increase	1.26	0.91	0.08
Effect = 0.05, 400% Cost increase	2.52	1.81	0.16

During the study, the full cost to the research programme to implement the study was 19,342 USD. Of these costs, 45% were costs of developing, pretesting and finalizing text-messages, 12% were costs of developing and testing of text-message distribution system, 29% were costs of collecting health workers' phone numbers, and only 13% were intervention implementation costs (sending messages and monitoring/trouble shooting the system to ensure message delivery). From Table 3, with an estimated 38,435 children correctly managed due to the intervention, the estimated cost per additional child correctly managed in study areas is 0.50 USD. Costs for Scenario 2 are estimated to be 28% less than for Scenario 1 (13,920 USD) due to lower costs of collecting health workers' phone numbers. From Table 3, the cost per additional child correctly managed under Scenario 2 would fall to 0.36 USD.

Table 2 summarizes costs if the intervention was scaled up to the national level (Scenario 3). In this case, costs are estimated to increase to 97,350 USD, the majority of which (66%) would be for sending 5.2 million text-messages to the 20,000 front-line health workers in public facilities. The remaining part of the costs would be required for refinement of the existing text-messages around the new case-management recommendations and their pre-testing in the field (20%) while only 8% would be needed to develop the messaging distribution system and 6% for generating the national list of health workers' phone numbers.

Table 3 also reports for each scenario how the cost per additional child correctly managed would change if the effect of the intervention was smaller or total costs of the implementation were larger. Economies of scale for implementing this intervention at the national level are shown clearly in Table 3. Because of the minor additional cost of text-messages for national scale-up (essentially 0.0124 USD per message), the cost per child correctly managed would fall to only 0.03 USD with estimated costs and the same effect size as found in the intervention study. Perhaps most importantly, even if the effect size was only 5% (meaning only 5% more children correctly managed due to the intervention during a national scale-up) or the cost of the intervention during national scale-up was 400% higher than estimated in Table 2, the cost per child correctly managed would only be 0.16 USD.

## Discussion

Simple and effective quality improvement interventions that can be rapidly scaled up within weak health systems in Africa remain urgently needed. We have shown that simple text-message reminders result in large, sustained improvements in paediatric malaria case-management [9] and that this innovative intervention was well accepted by health workers [10]. However equally important for policy makers is the feasibility of implementing the intervention as part of routine practice and the associated financial costs to the government implementing the intervention. Our cost-effectiveness analysis shows that under trial conditions, which are substantially higher that would be needed if implemented as routine practice, the cost per additional child correctly managed was only 0.5 USD. Moreover, we estimated that scale-up to the national level by the Ministry of Health would require a budget of about 98,000 USD and with a cost of only 0.03 USD per additional child correctly managed. Sensitivity analysis at the national level suggests that the intervention would remain highly cost-effective (0.16 USD per additional child correctly managed) even if the program only improved malaria case-management by 5 percentage points.

Very little evidence exists on the cost-effectiveness of interventions for improving adherence to malaria guidelines [14], [15]. We are aware of only one study with similar outcomes where effects of educational strategy on retailers' adherence to antimalarial dispensing standards were tested in Kenya [16]. The results of this study have shown 13 percentage points improvement in provider's adherence, with the cost per additional child correctly treated of 4.00 USD in the project area or 0.84 USD if intervention would be routinely implemented and expanded to the district level. Our analysis of routine implementation in the study district (Scenario 2) suggested a cost of only 0.36 USD per additional child correctly managed.

Importantly, an overall intervention cost on the national scale of 98,000 USD would represent only 1% of the 10 million USD awarded by The Global Fund to the Kenyan Ministry of Health to strengthen malaria case-management between 2011 and 2015 and therefore represents a reasonable and affordable expenditure. Availability of funding, the feasibility, effectiveness, and low cost of the intervention suggest that Kenya could consider national scale-up in the near future. In addition, the intervention can be viewed as a complement to, rather than a replacement of, existing programmes to strengthen malaria case-management.

Alongside the future scale-up, several questions with respect to text-messaging need to be addressed by further research. First, what is a reasonable frequency and duration of a text-message reminder intervention? Despite our findings that the intervention was not burdensome and was indeed well accepted by health workers, sending 2 messages daily for 5 days a week over 26 weeks to each health worker leaves limited space for other similar, non-malaria quality improvement interventions. Second, what is the effectiveness of the intervention around new “test and treat” malaria case-management policy as opposed to the presumptive policy tested under the trial conditions? Theoretical constructs suggest that reminders are mainly effective at the action and maintenance stages of behavior change, while they are less likely to be effective in changing practices that have not yet been accepted as clinical norms such as adherence to test negative results [10]. Third, would this intervention be equally effective in patients 5 years and older as it has been shown for young children? Older children and adults are the category of patients where non-adherence to guidelines has been frequently reported [17], [18], and with a decline of malaria transmission the correct management is becoming equally important. Fourth, despite encouraging findings that the intervention effects were sustained 6 months following termination of the intervention, longer term studies are required to confirm whether one-time delivery of the intervention presents an optimum mode of delivery or perhaps periodic delivery (e.g. every 2–3 years) would be required to maintain long-term effectiveness. Finally and perhaps most importantly, can we develop an effective text-message intervention addressing integrated management of the most common outpatient diseases? Such intervention may improve management of non-malaria conditions but would also facilitate adherence to malaria guidelines, e.g. health workers are more likely to adhere to test negative results if they are reminded of appropriate management of other conditions.

## Conclusions

This study provides the first cost-effectiveness analysis in the field of mobile health and adherence to malaria guidelines. We provided evidence that simple text-messaging intervention was indeed inexpensive under the study conditions with substantial economies to be gained if the intervention is deployed on larger scale. Further research is justified to optimize delivery and expand targets of the intervention.

## Supporting Information

Appendix S1 **Detailed assumptions for costs in** **Table 1** **and** **Table 2** **.**
(DOCX)
