# Supplementary material for: Costs and Cost-Effectiveness of a Mobile Phone Text-Message Reminder Programmes to Improve Health Workers' Adherence to Malaria Guidelines in Kenya
Source: PLoS One. 2012 Dec 18;7(12):e52045. doi: 10.1371/journal.pone.0052045 (PMC3525566; doi:10.1371/journal.pone.0052045)
Supplement: Appendix S1 — Detailed assumptions for costs in Table 1 and Table 2 . (DOCX) [file pone.0052045.s001.docx]

**Appendix S1. Detailed assumptions for costs in Table 1 and Table 2**

**SCENARIO 1**

**Development of intervention:** All costs for developing and refining the text-messages were personnel costs (1.A in Table 1). For each person implementing the intervention for each scenario, we first calculated the annual cost to the employer as the annual salary cost plus allowances and benefits (e.g., housing, health insurance, retirement) plus any taxes and other payments for employers (labor taxes, national insurance, etc). To estimate a full daily wage to each employee, we estimated an average of 216 actual working days per year based on a 5-day working week (365 days annually minus 104 weekend days, 10 national public holidays, 30 days annual leave, and 5 sick days). The full labor cost to the employer is then the annual salary cost divided by 216. Assuming an 8-hour working day, the full daily wage was then converted to hours as needed (for example, 2 hours for a research assistant was valued at 2/8ths of a daily wage). For this analysis, the annual average exchange rates of 127.37KES/GBP and 80.53 KES/USD for 2010 are used throughout ([www.oanda.com/currency/converter/](http://www.oanda.com/currency/converter/)). For privacy reasons, while level of effort information for each staff member is provided in Table 1, total labor costs for developing and refining the messages are reported in Tables 1 and 2. Exact details of these calculations for each staff member are not reported for privacy reasons.

Costs for pretesting messages are included in Section 1.B of Table 1. A flat fee of 40 KES/km is charged by the research programme for vehicle usage. The project traveled 5274.5 kms during this phase of the study. A research assistant and a driver traveled twice to the health facilities to pre-test messages with health workers at facilities not included in the study (20 working days for each person). Due to traveling over weekends, a total of 28 days of subsistence per-diem was provided (KES 2000 per day, 28 days, 2 people).

**Development of distribution system**: All costs are direct payments for the items listed (includes VAT).

**Costs of collecting health workers phone numbers:** A flat fee of 40 KES/km is charged for vehicle usage. The project traveled 5400 kms during the during the baseline health facility survey. A research assistant and a driver traveled to the health facilities to collect telephone numbers for the health workers at the facilities (20 working days each). Due to traveling over weekends, a total of 28 days of subsistence per-diem was provided (KES 2000 per day, 28 days, 2 people).

**Implementation costs.** Total cost of sending text messages is based on direct airtime costs. All costs for collecting and entering phone messages are included in the costs of collecting the health worker phone numbers. Monitoring and trouble shooting of the system was estimated to require 25% of full time research assistance over six months of sending the messages (28 days over six months at the full cost to the employer).

**SCENARIO 2**

All costs for Scenario 2 are the same as for Scenario 1 except the costs of collecting the health worker telephone numbers. This task could be completed by the District Public Health Nurse (DPHN). DPHN would call each facility in a district to update mobile phone numbers for health workers. He/she would then email the list to the DOMC implementing the program. Other than airtime and DPHN’s time to do this, estimated at 1 day per person per district, no other additional costs are incurred by this activity.

**SCENARIO 3**

**Development of intervention:** Scenario 3 assumes that the basic intervention messages would need to be revised substantially to be consistent with the new treatment guidelines. We assumed the same process would be followed here as during the intervention study but have used DOMC staff, external consultant’s support and employer costs to estimate the costs of developing and refining the intervention (Table 2, 1.A). Similar assumptions were made for pretesting messages with health workers. Government per-diem rates for staff are used for these estimates, which are substantially higher (KES 9,000) than for the staff during the intervention study (KES 2,000).

**Development of distribution system**: We obtained a quote from companies providing bulk SMS services in Kenya. The cost of 557,942 includes a set-up fee (52,142 KES), account administration fee (69,252 KES), an SMS mask fee (6,948 KES), system development and reporting fee (350,000 KES) and system maintenance fee (99,600 KES). A project computer is needed for maintaining the database, setting up scheduling of messages, and extracting reports from the provider on delivery details.

**Costs of collecting health workers phone numbers:**  These costs are the same as for Scenario 2 but expanded to all districts in Kenya.

**Implementation costs.** Total cost of sending text messages is included in implementation costs for Scenario 3. The bulk rate is 1 KES per message for the volume needed for the intervention. Monitoring and trouble shooting is included in the services provided for development of the distribution system.
